# Injury patterns, demographics, and post-treatment outcomes at Volta regional hospital, 2021–2025

**DOI:** 10.3389/fpubh.2026.1878285

**Published:** 2026-09-15

**Authors:** Forgive Awo Norvivor, Elijah Kwasi Peprah, Nathaniel Annang Armah, Millicent Boadiwa-Yirenkyi, Samuel Tetteh

**Affiliations:** 1 Department of Epidemiology and Biostatistics Fred N. Binka School of Public Health, University Health and Allied Sciences, Ho, Ghana; 2 Department of Environmental Health and Sanitation, School of Hygiene, Accra, Ghana; 3 Department of Occupational Therapy, School of Hygiene, Accra, Ghana; 4 Department of Health Sciences, University of Cape-Coast, Cape Coast, Ghana

**Keywords:** Ghana, hospital, injuries, morbidity, treatment outcomes

## Abstract

**Background:**

Injuries are a major public health concern worldwide, contributing significantly to morbidity, mortality, and disability across all age groups. They arise from diverse causes, including home accidents, road traffic crashes, occupational hazards, animal bites, burns, and interpersonal violence.

**Objective:**

This study examined the distribution and temporal trends of injuries, the socio-demographic characteristics of injured patients, and the treatment outcomes of injury-related admissions at the Volta Regional Hospital between 2021 and 2025.

**Methods:**

A retrospective descriptive study was conducted using hospital records. Data on patient age, sex, education, occupation, injury types, and treatment outcomes were extracted and presented in frequencies and percentages. Data was cleaned and analyzed using Stata version 17.

**Result:**

The results show that over the 5 years, home injuries were the leading cause of morbidity (68.8%−71.1% in the early years), Dog bites (up to 17.9%), burns (up to 11.6%), and snake bites (up to 3.9%) contributed moderately, while other causes remained below 5%. Among 449 patients for injury patients, most were male (72.5%), adults (37.2%), and had JHS/Middle School education (39.7%). Artisans constituted the largest occupational group (40.1%). Regarding outcomes, 85.3% were discharged, 10.5% transferred, 2.5% absconded, and 1.8% died.

**Conclusion:**

This study showed that injuries were more common among males and adults, with fractures, lacerations, and other trauma-related injuries accounting for most admissions at the Volta Regional Hospital between 2021 and 2025. Most patients were successfully discharged following treatment, although some required referral, absconded, or died. These findings highlight the need to strengthen injury prevention measures, trauma care services, and rehabilitation programs to improve patient outcomes and reduce the burden of injuries in the region.

## Background

Injuries are defined as physical harm or damage caused by external forces, whether intentional or unintentional, including accidents, falls, burns, road traffic collisions, and occupational hazards (1). Injuries constitute a major public health challenge contributing significantly to morbidity, mortality, disability, and economic burden (2). Globally, injuries account for over 5 million deaths annually, representing approximately 9% of all deaths, while non-fatal injuries are far more prevalent, often resulting in temporary or permanent disability, loss of productivity, increased healthcare costs, and psychosocial consequences for patients and their families (2). Road traffic injuries alone are responsible for an estimated 1.3 million deaths and 50 million non-fatal injuries annually, disproportionately affecting young adults aged 15–44 years, who are considered the most economically productive population segment, highlighting the societal and economic implications of trauma (2).

The burden of injuries is particularly high in Sub-Saharan Africa (SSA), where limited infrastructure, rapid urbanization, weak enforcement of safety regulations, and restricted access to emergency care contribute to poor injury outcomes (2). Road traffic crashes, falls, burns, and domestic injuries remain important causes of morbidity and mortality in the region, with children, older adults, and young adult males being disproportionately affected (3, 4). In Ghana, injuries contribute substantially to hospital admissions, with road traffic accidents, falls, burns, and occupational injuries among the most frequently reported causes (5). Injury-related morbidity and mortality continue to place a significant burden on individuals, families, and the healthcare system (3).

Despite the growing burden of injuries in Ghana, evidence on injury patterns, socio-demographic characteristics, and treatment outcomes remains limited in many parts of the country, particularly in the Volta Region. Hospital-based data are essential for understanding the epidemiology of injuries, identifying vulnerable populations, evaluating treatment outcomes, and informing injury prevention and trauma care strategies. However, few studies have comprehensively examined injury patterns and post-treatment outcomes using recent hospital records from the Volta Regional Hospital.

Therefore, this study assessed the socio-demographic characteristics, injury patterns, and treatment outcomes of patients presenting with injuries at the Volta Regional Hospital from 2021 to 2025. The findings are expected to provide evidence to support injury prevention initiatives, strengthen trauma care services, and inform health policy and planning in the region.

## Methods and material

### Study design

This study adopted a retrospective descriptive study design. A retrospective approach involves reviewing and analyzing existing hospital records and patient data over a specific period, in this case from 2021 to 2025. This design was appropriate for examining the distribution and trends of injuries, the demographic characteristics of patients, and the post-treatment outcomes, such as discharge status, mortality, transfers, and absconding rates. The descriptive cross-sectional design allowed for the summarization of data at specific points in time to provide a clear picture of injury patterns, identify high-risk populations, and highlight trends in patient outcomes. This design is particularly useful in hospital-based studies where the goal is to analyze existing clinical and administrative records to inform healthcare planning, improve patient management, and guide preventive strategies.

### Study site and population

The study was conducted at the hospital, a major referral healthcare facility located in the Volta Region of Ghana. The hospital serves as the principal regional hospital, providing comprehensive medical and surgical services, including emergency care, trauma management, orthopedic surgery, and specialized treatment for severe injuries. It caters to a wide population, including residents of both urban and rural areas within the region and surrounding districts, making it a representative site for understanding injury patterns and healthcare outcomes. The hospital is equipped with an emergency unit, surgical wards, and intensive care facilities, which handle a significant number of trauma and injury cases annually. It maintains detailed patient records, including information on demographics, diagnosis, treatment, and post-treatment outcomes, which allowed for accurate retrospective data collection for this study.

Additionally, the hospital is a training center for healthcare professionals, which means it follows standardized clinical protocols for injury management and record-keeping. Given its role as a regional referral facility, the hospital receives patients with a wide range of injury types, from minor injuries to severe trauma requiring specialized care. This diverse patient population provides an opportunity to examine not only the distribution and trends of injuries but also the effectiveness of hospital treatment protocols, patient outcomes, and resource utilization. The hospital's accessibility and structured record-keeping system made it an ideal study site for analyzing secondary data over the 5 years, thereby providing insights into injury epidemiology and healthcare delivery in the Volta Region.

### Sampling procedure

This study used a census sampling approach, that is, using all hospital records of patients who presented with injuries between 2021 and 2025. Since secondary data from existing hospital records were used, the study included all cases that met the inclusion criteria, which were patients of any age or sex admitted with any type of injury and whose records contained complete information on demographics, injury type, and treatment outcomes. Records that were incomplete or missing key variables, or those of patients treated on an outpatient basis without full documentation, were excluded. By using all eligible records, this approach minimized selection bias, ensured comprehensive coverage of the population, and provided a robust dataset for analyzing injury patterns, demographic characteristics, and post-treatment outcomes over the 5 years.

### Data collection procedure

Data for this study were collected retrospectively from hospital records of patients who were admitted with injuries between 2021 and 2025. A structured data extraction form was designed to systematically capture relevant information from patient files, admission registers, and discharge summaries. The form included variables such as patient demographics (age, sex, occupation, and residence), type and cause of injury, date of admission, length of hospital stay, and post-treatment outcomes, including discharge status, mortality, transfers, and absconding. Trained research assistants and the principal investigator reviewed the hospital records and extracted the required information, ensuring consistency and accuracy. Only records that contained complete information on the selected variables were included, while incomplete or missing records were excluded from the analysis. The data collection process involved cross-checking information from multiple sources within the hospital records to verify accuracy and reduce errors. Data were collected over a defined period, and all patient identifiers were removed to maintain confidentiality and ensure ethical compliance. The use of secondary data allowed for efficient analysis of a large dataset spanning 5 years, providing insights into injury patterns, affected demographics, and treatment outcomes without interfering with ongoing hospital operations. Overall, the data collection procedure ensured a systematic and reliable approach for capturing comprehensive information necessary to achieve the study objectives.

### Measuring variables

#### Outcome variable

The primary outcome variables for this study were related to injury outcomes among patients admitted to the hospital. The main outcome variable was post-treatment patient status, which was captured from hospital records and categorized into four groups: discharged, transferred, absconded, and deceased. Discharge status indicated patients who were successfully treated and released. Also, transfers referred to patients moved to other healthcare facilities for further management, whilst absconding indicated patients who left the hospital before completing treatment, and deceased captured patients who died during admission. These categories provided a clear measure of hospital treatment outcomes and allowed for analysis of patterns associated with demographic and clinical characteristics. The secondary outcome variable was the type of injury sustained, which was obtained from the hospital records and grouped into major categories, including road traffic injuries, falls, burns, occupational injuries, and other trauma-related injuries. Categorizing injuries allowed for the identification of the most common types of injuries treated and facilitated trend analysis over the 5-year period.

#### Explanatory variables

The explanatory or independent variables were selected based on existing literature and the information available in hospital records. These included age, sex, occupation, residence (urban or rural), and referral source. Other variables extracted included admission date, length of hospital stay, and comorbidities. These variables were used to examine associations with injury types and post-treatment outcomes, as well as to identify high-risk groups and factors influencing patient outcomes.

### Data management and analysis

The data collected from hospital records were first checked for completeness, consistency, and accuracy before entry into STATA version 17 for analysis. Records with missing or incomplete information on key variables such as patient demographics, type of injury, or treatment outcomes were excluded. Data cleaning procedures included coding categorical variables, labeling variables appropriately, and generating new variables where necessary to facilitate analysis. Descriptive statistics were used to summarize the data. Frequencies and percentages were calculated for categorical variables such as sex, occupation, type of injury, and post-treatment outcomes (discharge, mortality, transfers, and absconding). Continuous variables such as age and length of hospital stay were summarized using measures of central tendency (mean, median) and dispersion (standard deviation, interquartile range). Trend analysis was conducted to examine changes in the distribution of different injury types and patient outcomes over the 5-year study period. Graphs, tables, and charts were generated in STATA to visually represent injury patterns, demographic distributions, and outcomes. Cross-tabulations were performed to explore relationships between demographic factors and types of injuries or treatment outcomes. All findings were presented in a clear and interpretable format to support evidence-based conclusions and recommendations.

### Ethical approval

Ethical clearance was obtained from the Volta Regional Hospital. In addition, permission was sought from the data archivist of the Demographic and Health Survey (DHS) program in Ghana. In addition, before the data was collected, the DHS program followed the expected ethical protocols. This therefore covers the ethical considerations for the use of this data.

## Result and discussion

### Socio demographic characteristics

Table 1 presents the socio-demographic characteristics of the participants. The age distribution shows that, the older adults were 95 (21.1%), followed by 167 (37.1%) adults, and 61 (13.5%) adolescents. In terms of sex, males constituted 333 (74.2%), whereas females accounted for 116 (25.8%). Educational attainment indicates that 140 (31.1%) participants had completed JHS or middle school, while 104 (23.1%) had no formal education. Both primary and senior high school education each represented 95 (21.1%), and only 15 (3.3%) participants attained tertiary-level education. Occupational status revealed that artisans made up the highest group with 180 (40.0%), followed by students, 156 (34.7%). Public servants accounted for 54 (12.0%), while 59 (13.1%) were unemployed. Concerning injuries, fractures were the most common type reported, 167 (37.1%), followed by injury 148 (32.9%) and laceration 134 (29.8%; Table 1).

**Table 1:** Socio-demographic characteristics.

Variables	Frequency (*n* = 449)	Percentage (%)
**Age (years)**		
10–19 (Adolescent)	61	13.5
20–39 (Adult)	167	37.2
4–9 (Early childhood)	31	6.9
Months−3 (Infants and toddlers)	20	4.4
40–60 (Middle-aged adults)	75	16.0
Above 65 (older adult)	95	21.1
**Sex**		
Female	116	25.8
Male	333	74.2
**Educational level**		
JHS/middle school	140	31.1
None	104	23.1
Primary	95	21.1
Senior high school	95	21.1
Tertiary	15	3.3
**Occupational status**		
Students	156	34.7
Artisan	180	40.1
Public servant	54	12.0
Unemployed	59	13.1
**Injuries**		
Fracture	167	37.1
Injury	148	32.9
Laceration	134	29.8
**Outcome**		
Absconded	11	2.4
Died	8	1.7
Discharged	383	85.3
Transferred	47	10.4

### Distribution and trends of different types of injuries treated over the years

The results show that morbidity patterns changed significantly between 2021 and 2025, with home injuries dominating in 2021 (68.8%) and 2022 (71.1%), but declining sharply to 1.2% by 2025, while transport injuries reduced from 21.1% in 2021 to 6.8% in 2025 (Figure 1).

**Figure 1:**
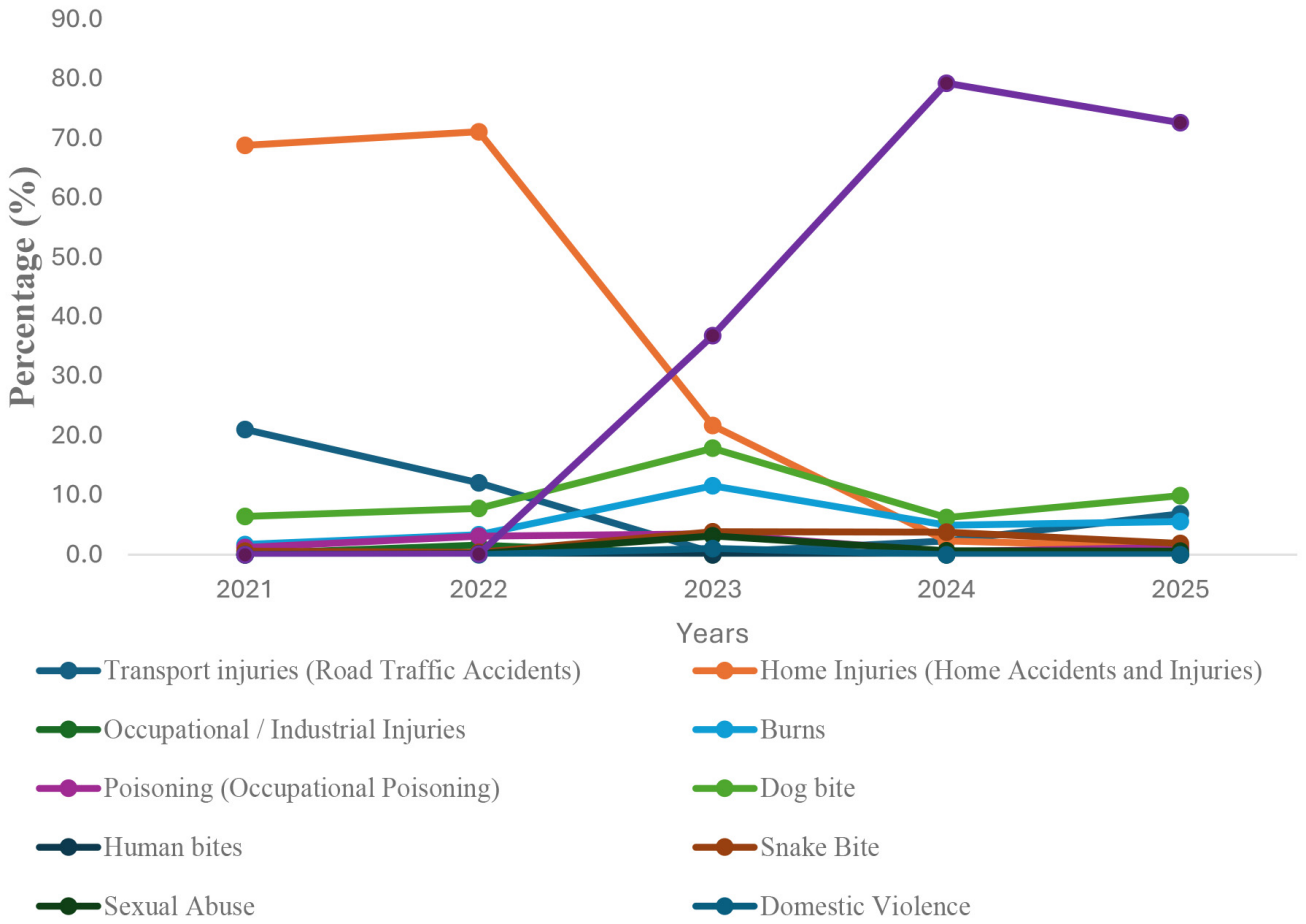
Distribution and trends of different types of injuries treated over the years.

### Demographic characteristics of patients with injuries

Table 2 presents the socio-demographic characteristics of patients with injuries. The study shows that adults accounted for 95 (26.9%), followed by middle-aged adults at 75 (21.2%) and adults at 87 (24.7%). Adolescents contributed 36 (10.2%), while young adults were 51 (14.4%). Early childhood cases were 31 (8.8%), pre-adolescents 25 (7.1%), and infants/toddlers 20 (5.7%). The older adult(s) group made up 29 (8.2%) of cases. In terms of sex distribution, the majority were male 333 (72.5%), while 116 (27.5%) were female. Regarding education, 140 (39.7%) had JHS/Middle School education, 96 (27.2%) had no formal education, 76 (21.5%) had primary education, 52 (14.7%) had Senior High School, and 15 (4.2%) attained tertiary level. Considering occupation, 167 (47.3%) were artisans, 134 (38.0%) were students, 54 (15.3%) were public servants, and 59 (16.7%) were unemployed (Table 2).

**Table 2:** Demographic characteristics of patients with types of injuries.

Variables	Patients presenting with injuries			Total Percentage (%)
	Fracture *n* (167)	Injury *n* (148)	Laceration *n* (134)	
**Age group**				
Adolescents	13	14	9	36 (8.0)
Adults	23	37	27	87 (19.4)
Early childhood	13	10	8	31 (6.9)
Older adult(s)	19	5	5	29 (6.5)
Infants and toddlers	6	5	9	20 (4.5)
Middle-aged adults	25	27	23	75 (16.7)
Older adults	44	27	24	95 (21.2)
Pre-adolescents	9	7	9	25 (5.6)
Young adults	15	16	20	51 (11.4)
**Sex**				
Female	46	32	38	115 (25.6)
Male	121	116	96	333 (74.2)
**Educational level**				
JHS/middle school	140	0	0	140 (31.2)
None	8	96	0	104 (23.2)
Primary	19	0	76	95 (21.2)
Senior high school	0	52	43	95 (21.2)
Tertiary	0	0	15	15 (3.4)
**Occupational status**				
Students	0	22	134	156 (34.7)
Artisan	167	13	0	180 (40.1)
Public servant	0	54	0	54 (12.0)
Unemployed	0	59	0	59 (13.1)
**Outcome**				
Absconded	2	2	7	11 (2.4)
Died	0	8	0	8 (1.8)
Discharged	132	130	121	383 (85.3)
Transferred	33	8	6	47 (10.5)

### Patient outcome after treatment

Figure 2 presents the treatment outcomes of patients with injuries. The results show that 383 (85.3%) were discharged, 47 (10.5%) were transferred for further management, 11 (2.5%) absconded, and eight (1.8%) died during the study period (Figure 2).

**Figure 2:**
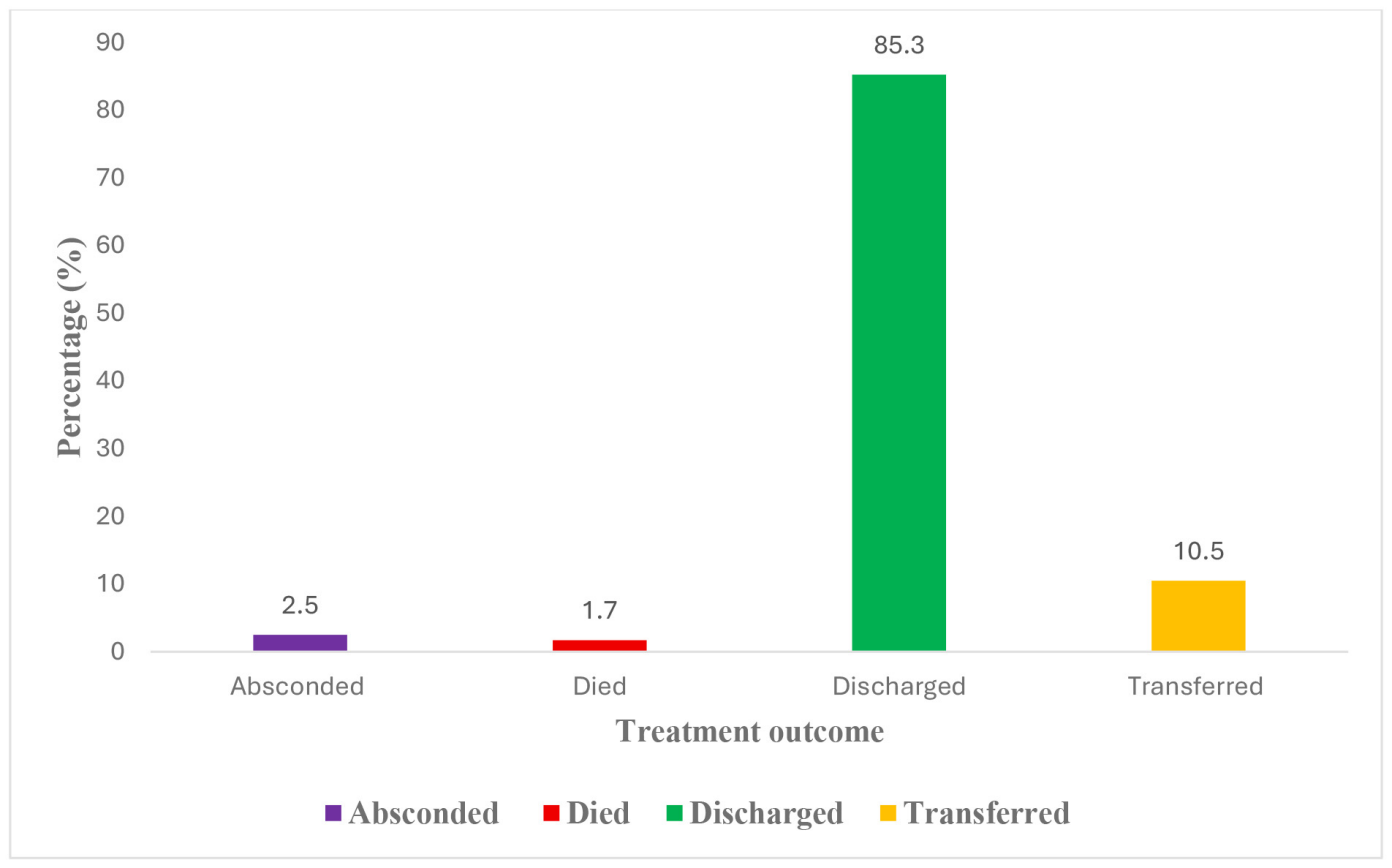
Patient outcome after treatment.

## Discussion

This study was a cross-sectional study with a quantitative approach using secondary data from the Volta region hospital, Ghana. This study assesses the pattern and distribution of injuries within 5 years, which is from 2021 to 2025.

### Distribution and trends of different types of injuries treated over injuries

This study explored the distribution and trends of morbidity due to injuries and related conditions over a 5-year period. The findings revealed that home injuries were the leading contributors in the earlier years (68.8–71.1%). Other conditions, such as dog bites (up to 17.9% in 2023), burns (11.6% in 2023), and snake bites (3.9% in 2023), played moderate roles, while occupational injuries, poisoning, sexual abuse, domestic violence, and human bites consistently contributed less than 5% each. These findings diverge from the global burden of injuries, where road traffic injuries (RTIs), falls, and violence are consistently reported as leading causes of injury-related morbidity and mortality [(2, 6, 14)]. RTIs alone account for 1.3 million deaths annually, particularly among individuals aged 5–29 years, making them the leading global cause of injury-related mortality. In contrast, RTIs were not observed as a major contributor in the present study, suggesting that context-specific exposures, household environments, and local health-seeking patterns may be shaping the epidemiology of morbidity in this setting.

The pre-dominance of home injuries in the early years aligns with reports from sub-Saharan Africa showing that children and older adults are particularly vulnerable to home-based accidents, such as falls and burns, due to age-specific risk factors and domestic hazards (7). Similar to findings from Tamale Teaching Hospital, Ghana, where falls and burns were prevalent among pediatric populations (7), the current study highlights the continuing role of household settings as critical sites of injury exposure.

Gyabaah et al. (7) noted burns as a recurrent cause of admission among children, which aligns with the moderate burden of burns found in this study. Interestingly, low contributions from occupational injuries, poisoning, and interpersonal violence contrast sharply with global and regional reports where occupational hazards, poisoning, and violence represent major concerns, particularly in LMICs with weak regulatory frameworks [(8, 14)]. This difference could be due to underreporting, stigma, or sociocultural factors that limit disclosure of sensitive causes such as domestic violence or sexual abuse.

#### Demographic characteristics of patients with injuries

The study revealed that injuries were most common among adults and middle-aged individuals, with adolescents and children also contributing a considerable proportion. This distribution aligns with global evidence that individuals in the 15–44 age group bear the highest burden of injury-related morbidity and mortality (9). Young and middle-aged adults are more exposed to road traffic accidents, occupational hazards, and interpersonal violence, which reflects the findings of Haagsma et al. (14) and Stevens et al. (8). The presence of pediatric and older adult(s) cases in the study is consistent with Gyabaah et al. (7), who reported high rates of burns and falls among children in Ghana, and with James et al. (6), who highlighted falls as the leading injury mechanism among older adults globally. Thus, the observed age distribution in this study reflects both international and local trends, emphasizing that while the economically active population remains most affected, children and the older adult(s) also constitute vulnerable groups requiring tailored prevention strategies.

The pre-dominance of male patients (72.5%) further confirms the widely documented gender disparities in injury epidemiology. WHO (2) estimates that men account for nearly three-quarters of injury-related mortality worldwide, a pattern attributed to higher occupational exposure, risk-taking behavior, and greater involvement in transport and outdoor activities. Studies from Africa, including Mwita et al. (17) in Tanzania and Osei et al. (16) in Ghana, have similarly shown that men, particularly those under 40, are disproportionately represented in trauma admissions. The findings of this study are therefore consistent with broader regional and global literature, suggesting that men, especially in their productive years, remain the most vulnerable group. Importantly, although women made up a smaller proportion, their injuries, often linked to domestic settings and violence, may be underrepresented due to underreporting, as highlighted by Gyabaah et al. (7).

Educational attainment among patients showed that the majority had either a middle school education or none at all. This reflects the strong association between lower educational levels and higher injury risk, as individuals with little or no education are often engaged in informal sector jobs lacking occupational safety protections. Donkor et al. (10) demonstrated that socio-economic deprivation, often linked with limited education, increases both the likelihood and severity of injury outcomes. Similarly, Mwita et al. (17) found that low-income men with minimal education were more vulnerable to hospital admissions for injuries. The current findings therefore reinforce the view that education plays a protective role in injury prevention by influencing occupational opportunities, risk awareness, and health-seeking behavior.

Occupational results revealed that artisans and students formed the largest groups among the injured. The high representation of artisans aligns with the structural realities of Ghana's labor market, where informal sector work pre-dominates. Artisans often work without adequate protective equipment or safety regulations, exposing them to risks of mechanical injuries, burns, and falls, consistent with findings in other African hospital-based studies (15). The significant number of students among the injured reflects the vulnerability of young people in school and recreational settings. (18) similarly observed that musculoskeletal injuries among adolescents in Nigeria were primarily sports-related, confirming that students face unique risks within and outside the school environment.

### Patient outcome after treatment

This study found that most trauma patients were successfully discharged (85.3%), indicating that the majority of injuries managed at Volta Regional Hospital were non-fatal and treatable within the facility's available resources. However, 10.5% of patients required transfer for further management, while 2.5% absconded and 1.8% died during the study period. These outcomes reflect both the strengths and systemic limitations of trauma care delivery in regional hospitals in Ghana. The high discharge rate aligns with findings from other sub-Saharan African trauma centers where fractures, lacerations, and moderate soft tissue injuries constitute the bulk of admissions and are generally associated with favorable in-hospital survival (15). However, the presence of transferred cases underscores the dependence of regional facilities on higher-level referral centers for definitive care, particularly for cases requiring advanced surgical, neurosurgical, or intensive care support. Stewart et al. (11) in Mozambique similarly reported high transfer rates, attributing them to the lack of critical care resources at district and regional hospitals. Such findings suggest that while primary stabilization is often achieved, systemic weaknesses in referral and transport pathways can delay definitive care, thereby affecting overall patient outcomes.

The mortality rate observed in this study (1.8%) is relatively low compared with reports from other African trauma hospitals where fatality rates often exceed 5% [(11, 19)]. This comparatively favorable outcome may be explained by two factors: first, the successful management of moderate injuries locally, and second, the transfer of more complex cases to higher-level facilities before in-hospital death occurs. However, literature warns that such low reported mortality may underestimate the true burden of trauma deaths, as patients who die in transit or at home after absconding are rarely captured in hospital statistics (12). The 2.5% absconding rate in this study is consistent with evidence from Ghana and other LMICs showing that financial barriers are a major determinant of pre-mature hospital departure. Osei et al. (16) reported that out-of-pocket healthcare costs frequently drive patients to leave against medical advice, undermining continuity of care. Such pre-mature exits increase the risk of untreated complications, infections, and long-term disability. This highlights the intersection of socio-economic vulnerability and clinical outcomes in trauma care, reinforcing the need for financial protection mechanisms to reduce catastrophic health expenditure.

Beyond immediate outcomes, the high discharge proportion should be considered within the broader context of long-term disability and rehabilitation needs. The Global Burden of Disease 2019 study estimated that musculoskeletal injuries alone account for over 90 million DALYs worldwide, disproportionately affecting LMICs (9). Yet rehabilitation access in sub-Saharan Africa remains critically low, with less than 20% of patients receiving needed physiotherapy or community-based rehabilitation after discharge (13). In Ghana, Amponsah-Bediako et al. (20) reported that many survivors of fractures experience persistent pain and reduced mobility due to inadequate follow-up care. This suggests that while discharge rates appear high in this study, many patients may continue to face long-term physical, psychological, and economic consequences that are not captured in routine hospital records. Taken together, these findings highlight that while most trauma patients at Volta Regional Hospital survive to discharge, post-treatment outcomes are shaped by systemic challenges such as referral dependence, financial barriers, and lack of rehabilitation services. Addressing these gaps will require strengthening critical care capacity at regional hospitals, improving referral systems, expanding access to rehabilitation, and reducing the financial burden on patients.

### Strengths and limitations of the study

#### Strengths

This study's main strength lies in its comprehensive analysis of injury patterns, socio-demographic characteristics, and patient outcomes over a 5-year period, providing robust evidence for public health planning and hospital management. The use of hospital records ensured reliable and objective data, allowing for the identification of temporal trends and high-risk groups, which is essential for targeting interventions. Inclusion of multiple injury types, age groups, and occupational categories enhanced the study's depth.

#### Limitations

The study is limited by its single-hospital setting, which may not fully represent injury patterns across other regions of Ghana, restricting generalizability. Its retrospective design relied on existing hospital records, which may be incomplete, inconsistent, or subject to recording bias. The analysis focused only on in-hospital outcomes, excluding long-term recovery, psychosocial consequences, and functional disabilities, thereby underestimating the true burden of injuries. Community-level injuries, minor cases, or patients who did not seek hospital care were not captured.

## Conclusion

This study showed that injuries were more common among males and adults, with fractures, lacerations, and other trauma-related injuries accounting for most admissions at the Volta Regional Hospital between 2021 and 2025. Most patients were successfully discharged following treatment, although some required referral, absconded, or died. These findings highlight the need to strengthen injury prevention measures, trauma care services, and rehabilitation programs to improve patient outcomes and reduce the burden of injuries in the region. To support SDG 3 on health and wellbeing, the Ghana Health Service should strengthen injury prevention programs, improve trauma care systems, and enhance follow-up and rehabilitation services to reduce morbidity and improve patient outcomes.

## Data Availability

The data analyzed in this study is subject to the following licenses/restrictions: it is sensitive patient data and it can be made available upon request through the research ethics committee via the email provided in the manuscript. Requests to access these datasets should be directed to University Of Health And Allied Sciences Research Ethics Committee (rec@uahs.edu.gh).
